# Diagnostic performance of machine learning models based on dual-phase ^**99**^mTc-MIBI SPECT/CT semiquantitative parameters for differentiating benign and malignant pulmonary nodules

**DOI:** 10.1371/journal.pone.0353271

**Published:** 2026-07-06

**Authors:** Kun Zhang, Xin Zhou, Yuhang Zhang, Gang Jin, Ping Li, Yuzhuo Xing

**Affiliations:** 1 Department of Nuclear Medicine, The Second Affiliated Hospital of Harbin Medical University, Nangang District, Harbin, Heilongjiang, China; 2 Department of General Surgery II, Heilongjiang Hospital of Beijing Children’s Hospital, Daoli District, Harbin, Heilongjiang, China; University of Messina Department of Biomedical Dental Morphological and Functional Imaging Sciences: Universita degli Studi di Messina Dipartimento di Scienze biomediche odontoiatriche e delle immagini morfologiche e funzionali, ITALY

## Abstract

**Purpose:**

To evaluate the diagnostic value of machine learning models based on dual-phase ^99^mTc-MIBI SPECT/CT semiquantitative parameters for differentiating benign and malignant pulmonary nodules.

**Methods:**

This retrospective study included 132 patients with pulmonary nodules, including 30 benign and 102 malignant lesions. All patients underwent dual-phase ^99^mTc-MIBI SPECT/CT at approximately 20 minutes and 2 hours after tracer injection. Semiquantitative parameters, including early and delayed tumor-to-normal ratios (T/N) and retention indices (RI), were calculated. Clinical variables and imaging parameters were analyzed using univariable and multivariable logistic regression, and selected variables were further used to develop machine learning models.

**Results:**

Malignant nodules showed significantly higher early-phase uptake and lower retention index values than benign nodules. Multivariable analysis identified elevated CEA and RImax as independent predictors of malignancy. Machine learning models built on these simple semiquantitative parameters showed promising diagnostic performance, with an AUC of 0.944 (95% CI: 0.883–0.990) for SVM on the training set, 0.805 (95% CI: 0.678–0.912) for Logistic Regression (LR), 0.881 (95% CI: 0.800–0.949) for Artificial Neural Network (ANN), and 0.979 (95% CI: 0.951–0.995) for Random Forest (RF), demonstrating their effectiveness in classifying pulmonary nodules.

**Conclusion:**

Dual-phase ^99^mTc-MIBI SPECT/CT semiquantitative parameters provide useful information for distinguishing benign from malignant pulmonary nodules. A machine learning strategy based on simple and interpretable parameters may offer a practical tool for pulmonary nodule assessment, especially in settings where complex imaging analysis is not feasible.

## Introduction

Lung cancer remains the leading cause of cancer-related death worldwide and continues to impose a substantial public health burden [1]. Early identification of malignant pulmonary nodules is therefore essential for timely treatment and improved prognosis [2,3]. In clinical practice, ^18^F-FDG PET/CT is widely used for the evaluation of pulmonary nodules and staging of lung cancer [4,5]. However, its use may be limited by cost, availability, and false-positive uptake in inflammatory or infectious lesions [6].

Compared with PET/CT, ^99^mTc-MIBI SPECT/CT is more widely available and less resource-intensive [7,8]. As a lipophilic cationic tracer, ^99^mTc-MIBI accumulates in mitochondria in relation to transmembrane potential and may also be affected by multidrug resistance-related efflux mechanisms. These biological properties make dual-phase MIBI imaging potentially useful for characterizing pulmonary nodules. Previous studies have suggested that semiquantitative indices derived from MIBI imaging, particularly tumor-to-normal ratios and retention-related parameters, may help distinguish malignant from benign lesions [9,10]. Nevertheless, the diagnostic performance of single parameters has been variable, and a more integrated analytical strategy may be needed.

Machine learning offers a practical approach for combining multiple variables and capturing nonlinear relationships among predictors [11,12]. In contrast to radiomics and deep learning, which often require complex segmentation, high-dimensional feature extraction, and larger sample sizes, a parameter-based machine learning strategy is simpler, more interpretable, and easier to implement in routine practice. For relatively small cohorts, this approach may also reduce the risk of overfitting while preserving clinical applicability.

Therefore, the present study aimed to develop and compare several machine learning models based on dual-phase ^99^mTc-MIBI SPECT/CT semiquantitative parameters and selected clinical variables for differentiating benign and malignant pulmonary nodules. We further sought to identify the most informative predictors and to evaluate whether a simplified parameter-based approach could provide robust diagnostic performance.

## Materials and methods

### Study population

This prospective study was conducted in accordance with the Declaration of Helsinki. Written informed consent was obtained from all participants before enrollment. The Medical Ethics Committee of the Second Affiliated Hospital of Harbin Medical University approved this retrospective study (Approval No. KY 2020−213). Patients with suspected pulmonary nodules or masses were consecutively recruited during two periods, from August to November 2022 and from February to April 2023. Dual-phase ^99^mTc-MIBI-SPECT/CT was performed under an ethics-approved clinical research protocol for pulmonary nodule assessment and was not conducted as a therapeutic clinical trial. The final diagnosis of all included pulmonary nodules was confirmed by pathological examination.

The inclusion criteria were as follows: (1) pulmonary nodule or mass with a diameter of at least 1 cm on imaging examinations; (2) no previous antitumor treatment; and (3) no history of other malignancies. The exclusion criteria were: (1) pulmonary nodules smaller than 1 cm or pure ground-glass nodules; (2) poor image quality or incomplete imaging data; and (3) history of radiotherapy or chemotherapy. The inclusion and exclusion workflow is presented in Fig 1.

**Fig 1:**
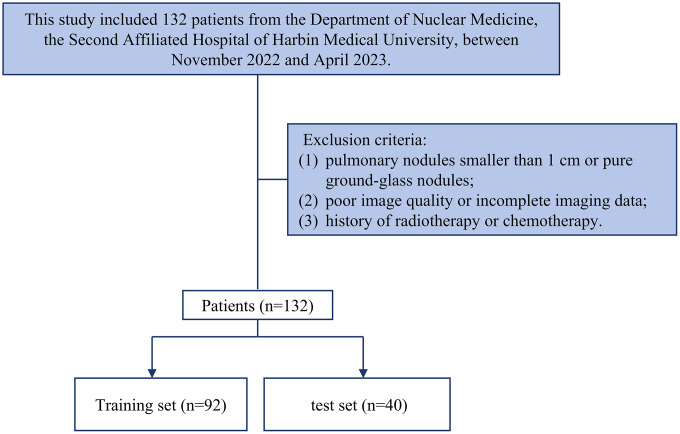
Flowchart of study population.

### Dual-phase ^99^mTc-MIBI SPECT/CT acquisition

All SPECT/CT examinations were performed using a Philips Precedence 6-slice hybrid SPECT/CT system (Philips Nuclear Medicine, Inc., Milpitas, CA, USA) equipped with low-energy high-resolution collimators. The sestamibi kit was purchased from Jiangyuan Pharmaceutical Factory of Wuxi Jiangyuan Industrial Technology and Trade Co., Ltd. (Wuxi, China).. After intravenous administration of 740–1110 MBq of ^99^mTc-MIBI, early thoracic imaging was acquired at approximately 20 min after injection, followed by delayed imaging at 2 h. All studies were performed on a hybrid SPECT/CT system equipped with low-energy, high-resolution collimators. The acquisition field of view covered only the thoracic/chest region, including the pulmonary lesion and adjacent thoracic structures.

SPECT acquisition was performed in step-and-shoot mode using a 128 × 128 matrix, 64 projections per detector over 180°, and 20 s per projection. The acquisition time of 20 s per projection was selected according to the standard imaging protocol of our center, aiming to obtain sufficient count statistics and acceptable image quality while avoiding an unnecessarily long acquisition time for patients. CT was obtained for attenuation correction and anatomical localization using a standard-resolution protocol with 120 kV tube voltage, 300 mAs per slice, 6 × 1.5 mm collimation, 0.9 pitch, 0.75 s rotation time, 350 mm field of view, 512 × 512 matrix, and reconstructed slice thickness of 2 mm with a 1-mm increment. Fused SPECT/CT images were generated automatically by the workstation.

### Semiquantitative parameter measurement

For semiquantitative analysis, regions of interest (ROIs) were manually placed on fused SPECT/CT images to encompass the lesion. A reference ROI of the same size was positioned in normal lung parenchyma on the contralateral side; when appropriate, ipsilateral normal lung tissue was used as the reference. Maximum and mean counts were recorded for both early and delayed phases.

The following parameters were calculated:

- T/Nmax,Early
- T/Nmean,Early
- T/Nmax,Delayed
- T/Nmean,Delayed
- RImax
- RImean

The tumor-to-normal ratio (T/N) was defined as the ratio of lesion counts to background lung counts. The retention index (RI) was defined as the relative percentage change between delayed and early uptake.

### Clinical variables

Clinical variables collected for analysis included age, sex, smoking history, serum carcinoembryonic antigen (CEA), and squamous cell carcinoma antigen (SCC), according to data completeness and clinical relevance.

### Feature selection and model development

Continuous variables were standardized before model construction, and categorical variables were encoded as appropriate. The dataset was randomly divided into a training set and a test set at a ratio of 7:3.

Feature selection was performed before machine learning modeling. Variables showing significant differences between benign and malignant nodules were first identified as candidate predictors through univariable analysis. Collinearity among variables was then evaluated using correlation analysis, and redundant variables were excluded. Subsequently, multivariable binary logistic regression analysis was performed to identify independent predictors of malignancy. Variables that remained significant or showed clear clinical relevance were finally selected as inputs for machine learning model development.

Four machine learning algorithms were developed and compared:

1. Logistic regression (LR)
2. Support vector machine (SVM)
3. Random forest (RF)
4. Artificial Neural Network (ANN)

Hyperparameters were tuned in the training set using cross-validation. Model performance was assessed in the test set.

### Statistical analysis

The distribution of continuous variables was assessed using the Shapiro-Wilk test. Normally distributed variables were expressed as mean ± standard deviation and compared using the independent-samples t test, whereas non-normally distributed variables were expressed as median and interquartile range and compared using the Mann-Whitney U test. Categorical variables were compared using the chi-square test or Fisher’s exact test, as appropriate.Model performance was evaluated using receiver operating characteristic (ROC) curves and AUCs. Sensitivity, specificity, accuracy, and F1-score were calculated using the optimal cutoff determined by the Youden index in the training set. DeLong’s test was used to compare ROC curves when applicable. A two-sided P value < 0.05 was considered statistically significant.

## Results

### Patient characteristics and semiquantitative parameter differences

The baseline clinical characteristics and semiquantitative imaging parameters of the patients are summarized in Table 1. A total of 132 patients were included in the analysis, comprising 30 benign and 102 malignant pulmonary nodules.

**Table 1:** Clinical characteristics of the patients.

Variables	Overall(n = 132)^1^	Training set(n = 92)^1^	Test set(n = 40)^1^	*p*-value^2^
Pathology				>0.999
Benign	30.0 (22.7%)	21.0 (22.8%)	9.0 (22.5%)	
Malignant	102.0 (77.3%)	71.0 (77.2%)	31.0 (77.5%)	
Gender				0.877
Female	69.0 (52.3%)	49.0 (53.3%)	20.0 (50.0%)	
Male	63.0 (47.7%)	43.0 (46.7%)	20.0 (50.0%)	
Smoking	68.0 (51.5%)	47.0 (51.1%)	21.0 (52.5%)	>0.999
CEA				0.226
<5	65.0 (49.2%)	49.0 (53.3%)	16.0 (40.0%)	
≥5	67.0 (50.8%)	43.0 (46.7%)	24.0 (60.0%)	
SCC				0.569
<1.5	117.0 (88.6%)	83.0 (90.2%)	34.0 (85.0%)	
≥1.5	15.0 (11.4%)	9.0 (9.8%)	6.0 (15.0%)	
Age	59.0 [56.0, 62.0]	59.0 [56.0, 62.0]	59.0 [57.5, 64.5]	0.261
TNmean_Early	1.2 [1.0, 1.6]	1.3 [1.0, 1.7]	1.2 [0.9, 1.4]	0.169
TNmax_Early	1.3 [1.1, 1.9]	1.3 [1.1, 1.9]	1.3 [1.0, 1.9]	0.729
TNmean_Delayed	1.3 [1.0, 1.6]	1.3 [1.0, 1.6]	1.4 [1.0, 1.9]	0.488
TNmax_Delayed	1.5 [1.1, 2.0]	1.5 [1.1, 1.9]	1.5 [1.1, 2.5]	0.513
RImean	3.7 [−21.3, 32.1]	−2.5 [−26.9, 25.6]	12.6 [−9.5, 41.3]	0.043
RImax	8.9 [−18.0, 42.0]	3.3 [−22.0, 59.9]	10.8 [−12.4, 27.4]	0.440

^1^n (%); Median [Q1, Q3].

^2^Pearson’s Chi-squared test; Kruskal-Wallis rank sum test.

Abbreviations: CEA, carcinoembryonic antigen; SCC, squamous cell carcinoma antigen; T/N, tumor-to-normal ratio; RI, retention index.

The clinical variables, including gender, smoking history, CEA, SCC, and age, showed no significant differences across the groups (p > 0.05). In terms of semiquantitative parameters derived from dual-phase ^99^mTc-MIBI SPECT/CT, the early-phase tumor-to-normal ratios (T/Nmean, Early and T/Nmax, Early) did not show significant differences between the overall cohort, training set, and test set (p = 0.169 and p = 0.729, respectively). Similarly, delayed-phase T/N ratios (T/Nmean, Delayed and T/Nmax, Delayed) did not differ significantly (p = 0.488 and p = 0.513). However, RImean exhibited a significant difference between the groups (p = 0.043), suggesting it could be a critical parameter for model development, while RImax did not show a significant difference (p = 0.440). These findings highlight that while certain clinical and semiquantitative parameters remain stable across the training and test sets. In addition, elevated CEA levels (≥5 ng/mL) were significantly more common in malignant nodules than in benign nodules (57.84% vs 26.67%, P = 0.003).

### Logistic regression analysis

The results of the univariable and multivariable logistic regression analyses of the clinical characteristics are presented in Table 2. Univariable analysis identified several candidate variables associated with malignancy, including early-phase T/N parameters, RI-related indices, and CEA level. In the multivariable logistic regression model, elevated CEA remained an independent predictor of malignant pathology. Among the imaging-derived parameters, RImax also retained independent significance and showed an inverse association with malignancy. Other candidate variables did not remain statistically significant after adjustment.

**Table 2:** Results of logistic regression analysis for clinical characteristics.

Characteristic (reference level)	Univariate analysis			Multivariable analysis		
	OR	95 CI	*p*-value	OR	95 CI	*p-*value
Age	1.04	0.97-1.10	0.262			
Sex (Female)	0.75	0.33-1.69	0.485			
Smoking (Yes)	0.44	0.19-1.04	0.062			
CEA (≥5)	3.77	1.53-9.28	0.004*	4.69	1.43-15.35	0.011
SCC (≥1.5)	1.20	0.32-4.57	0.789			
T/Nmean Early	3.50	1.46-8.40	0.005*	4.00	0.90-17.82	0.069
T/Nmax Early	18.29	5.04-66.35	<0.001*	3.25	0.62-16.90	0.162
T/Nmean Delayed	1.60	0.73-3.49	0.236			
T/Nmax Delayed	1.12	0.70-1.79	0.641			
RImean	1.00	1.00-1.00	0.118			
RImax	0.99	0.98-0.99	<0.001*	0.99	0.98-0.99	0.007

Data in parentheses are reference level;

* Represents *p* < 0.05. OR, odds ratio; CI, confidence interval; LN, lymph node.

The final regression findings indicate that parameter-based risk assessment can be anchored by both biological information from serum tumor markers and kinetic information derived from dual-phase MIBI imaging.

### Performance of machine learning models

The Table 3 presents the performance evaluation of four different models across training and test datasets. Various performance metrics, including accuracy, sensitivity (True Positive Rate – TPR), specificity (True Negative Rate – TNR), precision (Positive Predictive Value – PPV), F1 score, and area under the ROC curve (AUC), are used to assess the performance of each model. Additionally, the 95% confidence intervals (CI) for the AUC values are provided. The predictive efficacy of these models is presented in Fig 2. Fig 3 shows the confusion matrices of the different models.

**Table 3:** Performance metrics of the machine learning models in the training and test sets.

Model	Accuracy	Sensitivity	Specificity	Precision	F1 Score	AUC (95% CI)
SVM (Train)	0.946	0.972	0.857	0.958	0.965	0.944 (0.883-0.990)
SVM (Test)	0.85	0.968	0.444	0.857	0.909	0.799 (0.620-0.936)
LR (Train)	0.717	0.69	0.81	0.925	0.79	0.805 (0.678-0.912)
LR (Test)	0.725	0.742	0.667	0.885	0.807	0.792 (0.603-0.945)
ANN (Train)	0.761	0.732	0.857	0.945	0.825	0.881 (0.800-0.949)
ANN (Test)	0.75	0.742	0.778	0.92	0.821	0.781 (0.503-0.989)
RF (Train)	0.924	0.901	1.0	1.0	0.948	0.979 (0.951-0.995)
RF (Test)	0.775	0.839	0.556	0.867	0.852	0.853 (0.694-0.969)

Abbreviations: SVM, support vector machine; LR, logistic regression; ANN, artificial neural network; RF, random forest; AUC, area under the receiver operating characteristic curve; CI, confidence interval.

**Fig 2:**
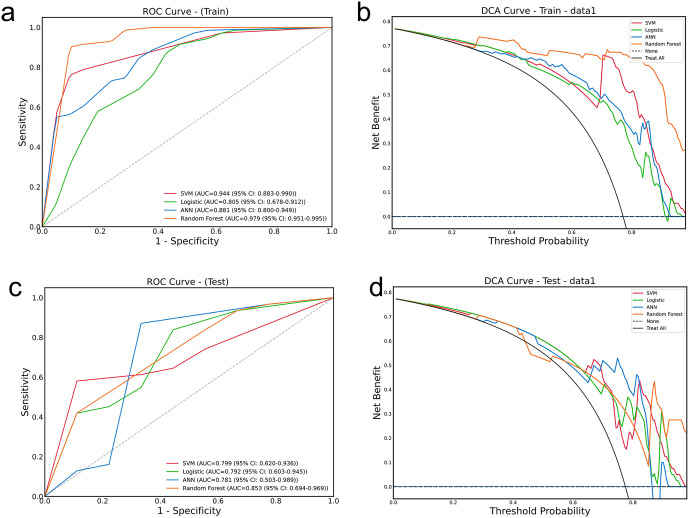
ROC curves and performance metrics of various models across different datasets. (A) ROC curve of the training set; (B) DCA curve of the e training set;(C) ROC curve of the test set; (D) DCA curve of the test set.

**Fig 3:**
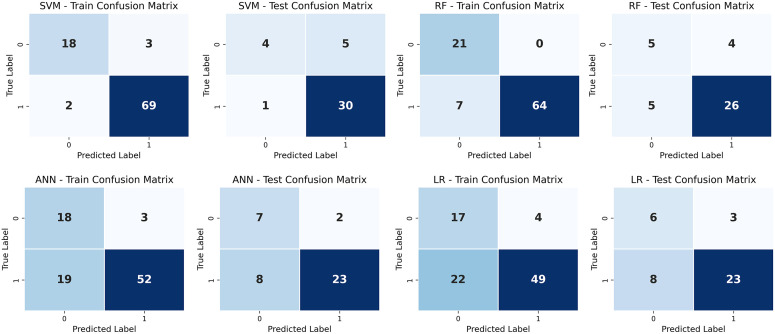
Confusion matrices of different models.

- SVM performed excellently on the training set with an accuracy of 94.6% and AUC of 0.944 (95% CI: 0.883–0.990). However, its performance on the test set was slightly weaker, with an accuracy of 85.0% and AUC of 0.799.
- LR showed moderate performance, with an AUC of 0.805 (95% CI: 0.678–0.912) on the training set. The test set performance was similar, with an AUC of 0.792, suggesting the model’s limited adaptability to new data.
- ANN demonstrated good performance on the training set, with an AUC of 0.881 (95% CI: 0.800–0.949). However, its performance on the test set dropped to an AUC of 0.781 (95% CI: 0.503–0.989), indicating relatively large generalization error.
- RF showed outstanding performance on the training set, with an AUC of 0.979 (95% CI: 0.951–0.995). While its performance decreased on the test set, with an AUC of 0.853, it still exhibited strong classification ability compared to other models.

Additionally, DeLong tests were conducted to assess the statistical significance of the differences between the models’ AUC scores. For the training set, the DeLong test results indicated statistically significant differences in the AUC values across the models, with SVM and RF showing the best overall performance. Similarly, the DeLong test results for the test set showed notable differences in AUC, with SVM achieving the highest AUC, followed by RF. These results underscore the importance of careful model selection based on both performance metrics and statistical validation. Two specific examples (Example text for the main manuscript) are shown in Fig 4.

**Fig 4:**
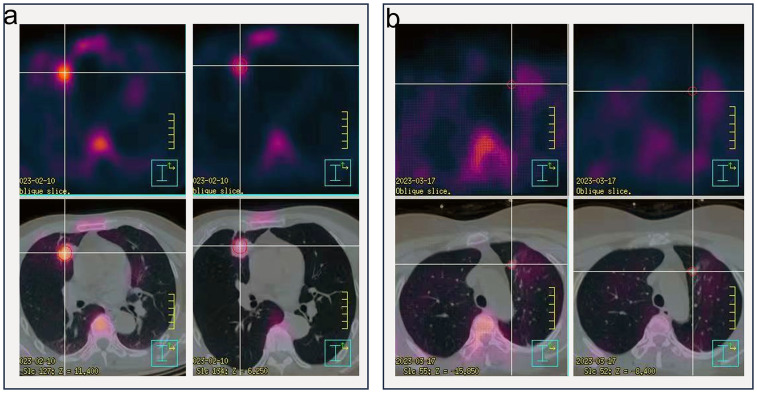
Representative cases of dual-phase^99^mTc-MIBI SPECT/CT imaging in pulmonary nodules. (a) A 61-year-old male patient showed markedly increased early uptake (RI_max = –80.73; T/N_mean-early = 4.10). (b) A 51-year-old female patient exhibited relatively low early uptake (RI_max = 185.4; T/N_mean-early = 1.10).

## Discussion

The present study explored a simplified machine learning strategy based on dual-phase ^99^mTc-MIBI SPECT/CT semiquantitative parameters for pulmonary nodule classification. Several important findings emerged. Our findings are broadly consistent with previous studies suggesting that ^99^mTc-MIBI imaging has potential value in differentiating benign from malignant pulmonary nodules. Earlier studies reported that both visual assessment and semiquantitative analysis of dual-phase ^99^mTc-MIBI imaging could provide useful diagnostic information for solitary pulmonary nodules [9,13,14].

In thyroid nodule assessment, persistent or increased delayed ^99^mTc-MIBI uptake is often considered suspicious for malignancy [15]. However, the kinetic behavior of ^99^mTc-MIBI in pulmonary nodules may differ from that in thyroid nodules. Pulmonary lesions are influenced by heterogeneous tumor biology, inflammatory changes, vascularity, mitochondrial activity, and multidrug resistance-related efflux mechanisms. In the present cohort, malignant pulmonary nodules tended to show higher early uptake but lower retention index values, suggesting that early tracer accumulation and delayed washout may provide complementary information. Therefore, the interpretation of dual-phase ^99^mTc-MIBI SPECT/CT in pulmonary nodules should not simply follow thyroid nodule criteria, but should consider lesion-specific tracer kinetics.

First, malignant nodules showed significantly higher early-phase MIBI uptake than benign nodules. This observation is biologically plausible because malignant cells usually exhibit increased perfusion, higher cellular density, and enhanced mitochondrial activity, all of which may contribute to greater initial tracer accumulation [16]. In our cohort, both T/Nmean,Early and T/Nmax,Early were significantly elevated in malignant lesions, indicating that early uptake remains an informative marker of malignancy.

Second, malignant nodules exhibited lower RI values, particularly RImax, which also remained independently associated with malignancy in multivariable analysis. Previous lung cancer studies have reported correlations between ^99^mTc-MIBI uptake or washout and P-glycoprotein expression, suggesting that tracer retention may be influenced by multidrug-resistance-related efflux mechanisms [17,18].This pattern suggests that delayed retention is not always greater in malignant lesions and that washout kinetics may vary according to lesion biology. One possible explanation is that the distribution of MIBI within pulmonary nodules is influenced not only by mitochondrial membrane potential but also by multidrug resistance-related efflux transport and local microenvironmental factors. Inflammatory or hyperplastic benign lesions may occasionally show persistent delayed uptake, whereas some malignant lesions may demonstrate relatively rapid washout. Therefore, early uptake and delayed retention should be interpreted jointly rather than separately. Another important finding of this study was that retention-related parameters, especially RImax, were associated with malignancy. This is in line with earlier evidence showing that ^99^mTc-MIBI washout kinetics may reflect biological characteristics beyond simple tracer uptake intensity [18].

Third, serum CEA was identified as an independent predictor of malignancy, supporting the idea that combining imaging-derived kinetics with routine clinical biomarkers can improve risk stratification. Rather than relying on a single imaging index, parameter-based machine learning allows complementary information to be integrated into a unified predictive framework.

An important advantage of the present work is its emphasis on simplicity and interpretability. Compared with radiomics and deep learning approaches, the current strategy does not depend on high-dimensional feature extraction or complex image processing pipelines. This may be particularly relevant for studies with limited sample sizes and for institutions seeking a practical diagnostic model that can be implemented without advanced computational infrastructure. From a methodological perspective, a low-dimensional feature space may also reduce instability and improve reproducibility.

The clinical significance of this work lies in the possibility of establishing a functional imaging-based classification tool that is more accessible than FDG PET/CT and easier to implement than radiomics or deep learning. In selected patients with indeterminate pulmonary nodules, especially in institutions where ^18^F-FDG PET/CT is unavailable, less accessible, or not routinely performed, semiquantitative^99^mTc-MIBI SPECT/CT parameters may provide additional functional information for risk stratification. The proposed machine learning model may help integrate simple imaging-derived kinetic parameters and clinical markers into a more interpretable assessment framework. In renal masses, for example, ^99^mTc-MIBI SPECT/CT has shown value in differentiating benign oncocytomas from renal cell carcinoma, highlighting the close relationship between tracer uptake and tumor mitochondrial content [19]. Similarly, in thyroid imaging, meta-analytic evidence has shown that ^99^mTc-MIBI may provide diagnostic information for differentiating benign from malignant nodules, although its performance should be interpreted cautiously [20].

This study has several limitations. First, it was conducted at a single center with a relatively small and imbalanced sample, which may limit generalizability. Second, although the feature space was intentionally simplified, internal validation alone is insufficient to establish model robustness, and external validation is still required. Third, the study focused on semiquantitative imaging parameters and selected clinical indicators; morphological CT features, radiomics features, and pathological subtype-specific analyses were not incorporated. Finally, the current manuscript framework assumes comparison across several machine learning algorithms, but the final interpretation should be based on the actual test-set results after model training is completed.

## Conclusion

Dual-phase ^99^mTc-MIBI SPECT/CT semiquantitative parameters provide meaningful information for differentiating benign and malignant pulmonary nodules. Malignant lesions were characterized by higher early tumor-to-normal uptake and lower retention index values, while elevated CEA and RImax emerged as independent predictors of malignancy. A machine learning strategy based on simple and interpretable parameters showed preliminary diagnostic potential in this single-center retrospective cohort. However, these findings require further validation in larger, multicenter studies before clinical application.
