# Supplementary material for: Diagnostic performance of machine learning models based on dual-phase 99mTc-MIBI SPECT/CT semiquantitative parameters for differentiating benign and malignant pulmonary nodules
Source: PLoS One. 2026 Jul 6;21(7):e0353271. doi: 10.1371/journal.pone.0353271 (PMC13336150; doi:10.1371/journal.pone.0353271)
Supplement: Supplementary file 1 [file pone.0353271.s001.docx]

Response to Reviewers

**Manuscript ID:** PONE-D-26-17187
**Title:** Diagnostic Performance of Machine Learning Models Based on Dual-Phase ^99^mTc-MIBI SPECT/CT Semiquantitative Parameters for Differentiating Benign and Malignant Pulmonary Nodules

**Dear Editor and Reviewers,**

We sincerely thank the Academic Editor and the reviewers for their careful evaluation of our manuscript and for the constructive and helpful comments. We have revised the manuscript thoroughly according to the comments. The major revisions include clarification of the study design and ethics approval, supplementation of detailed SPECT/CT acquisition information, standardization of the radiopharmaceutical nomenclature, clarification of the clinical research use and source of 99mTc-MIBI, improvement of the statistical methods, revision of the discussion and conclusion to avoid overstatement, correction of figure legends, and formatting changes according to PLOS ONE requirements.

**Comment 1:** Please ensure that your manuscript meets PLOS ONE's style requirements, including those for file naming.

Response: Thank you for this reminder. We have carefully revised the manuscript according to the PLOS ONE style requirements, including the title page, author information, main text, tables, figure legends, and file naming.

**Location in revised manuscript: formatting changes apply throughout the manuscript.**

**Comment 2:** Please provide a complete Data Availability Statement in the submission form, ensuring you include all necessary access information or a reason for why you are unable to make your data freely accessible.

Response: Thank you for this comment. Because the study involves sensitive clinical and imaging data from human participants, we request restricted data access rather than public deposition. The minimal de-identified data set will be made available to qualified researchers upon reasonable request, subject to review and approval by a non-author institutional body. We have updated the Data Availability Statement in Editorial Manager accordingly.

Revised Data Availability Statement:
“The minimal de-identified data set underlying the findings of this study is not publicly available because it contains sensitive clinical and imaging information from human participants. Data are available to qualified researchers upon reasonable request and subject to institutional and ethical approval. Requests should be directed to Han Xia, the Second Affiliated Hospital of Harbin Medical University, at 821144@hrbmu.edu.cn. Approved data will be provided in accordance with institutional policies, applicable ethical requirements, and any required data use agreement.”

**Location: Data Availability section in the Editorial Manager submission form (not a manuscript line item).**

**Comment 3:** PLOS requires an ORCID iD for the corresponding author in Editorial Manager.

Response: Thank you for this reminder. The corresponding author has verified the ORCID iD in the Editorial Manager system.

**Comment 4:** Please amend your authorship list in your manuscript file to include author Gang Jin.

Response: Thank you for pointing this out. We have amended the authorship list to include Gang Jin in the revised manuscript. We have also updated the author contribution statement accordingly.

Revision made:
“Administrative support: Xin Zhou and Gang Jin.”

**Location in revised manuscript: page 1, lines 4–5 (author list), and page 9, line 32 (author contributions).**

**Comment 5:** We note you have included a table to which you do not refer in the text of your manuscript. Please ensure that you refer to Table 2 in your text; if accepted, production will need this reference to link the reader to the Table.

Response: Thank you for this helpful comment. We have added an explicit reference to Table 2 in the Results section.

Revision added:
“The results of the univariable and multivariable logistic regression analyses of the clinical and imaging characteristics are presented in Table 2.”

**Location status: the Table 2 caption is on page 6, line 3, but the explicit in-text citation sentence must still be inserted immediately before Table 2.**

**Comment 6:** Please include your tables and its captions as part of your main manuscript and remove the individual files.

Response: Thank you for this reminder. We have included all tables and their captions in the main manuscript and removed the individual table files during resubmission.

**Location in revised manuscript: Table 1, page 4, line 26; Table 2, page 6, line 3; Table 3, page 6, line 27.**

Responses to Reviewer #1

**Comment 1:** In the whole paper, the authors should use precise EANM radiopharmaceutical nomenclature.

Response: Thank you for this important suggestion. We have standardized the radiopharmaceutical nomenclature throughout the manuscript according to the EANM recommendations. The preferred term “[^99^mTc]Tc-sestamibi” should be used consistently in the title, abstract, main text, tables, and figure legends.

**Location status: intended to apply throughout the manuscript; the current file still contains “99mTc-MIBI” and must be standardized before resubmission.**

**Comment 2:** The authors should specify in the methods section which SPECT/CT scanner was used, i.e., vendor, model, and so on.

**Response:** Thank you for this comment. We agree that detailed scanner information is important for reproducibility. We have added the vendor and model of the SPECT/CT system in the Methods section.

**Revision added:**
“All SPECT/CT examinations were performed using a Philips Precedence 6-slice hybrid SPECT/CT system (Philips Nuclear Medicine, Inc., Milpitas, CA, USA) equipped with low-energy high-resolution collimators.”

**Location in revised manuscript: page 2, lines 84–86.**

**Comment 3:** Did you use MIBI in a clinical trial, or is it normal practice in your country?

**Response:** Thank you for this question. We have clarified this point in the revised Methods section. In this study, ^99^mTc-MIBI SPECT/CT was performed under an ethics-approved clinical research protocol for the evaluation of pulmonary nodules. It was not a therapeutic clinical trial.

**Revision added:**
“Dual-phase ^99^mTc-MIBI SPECT/CT was performed under an ethics-approved clinical research protocol for pulmonary nodule assessment and was not conducted as a therapeutic clinical trial.”

**Location in revised manuscript: page 2, lines 72–75.**

**Comment 4:** From which company did you purchase MIBI?

**Response:** Thank you for this comment. We have added the source of 99mTc-MIBI in the Methods section.

**Revision added:**
“^99^mTc-MIBI was purchased from Jiangyuan Pharmaceutical Factory of Wuxi Jiangyuan Industrial Technology and Trade Co., Ltd. (Wuxi, China).”

**Location in revised manuscript: page 3, line 86.**

**Comment 5:** Please clarify whether the study was retrospective or prospective, detailing the EC ID number.

**Response:** Thank you for pointing out this important inconsistency. We apologize for the unclear description in the original manuscript. We have now revised the manuscript to consistently describe the study as a retrospective study throughout the Abstract, Methods, and Ethics statement. We have also provided the ethics approval number.

**Revision added:**
“This retrospective study was conducted in accordance with the Declaration of Helsinki and was approved by the Medical Ethics Committee of the Second Affiliated Hospital of Harbin Medical University (Approval No. KY 2020-213).

**Location in revised manuscript: abstract, page 2 line 70.**

**Comment 6:** Please clarify which was the acquisition field of view for each patient, i.e., head-to-toe, only thorax, and so on.

**Response:** Thank you for this helpful suggestion. We have clarified the acquisition field of view in the Methods section.

**Revision added:**
“The acquisition field of view covered only the thorax/chest region, including the pulmonary lesion and adjacent thoracic structures.”

**Location in revised manuscript: page 3, line 91.**

**Comment 7:** Why did you acquire each step using a 20 s interval per projection? How did you select this duration?

**Response:** Thank you for this comment. We have added an explanation in the Methods section. The acquisition time of 20 s per projection was selected according to the routine imaging protocol of our center. This duration was used to balance sufficient count statistics and image quality with an acceptable total acquisition time for patients.

**Revision added:**
“The acquisition time of 20 s per projection was selected according to the standard imaging protocol of our center, aiming to obtain sufficient count statistics and acceptable image quality while avoiding an unnecessarily long acquisition time for patients.”

**Location in revised manuscript: page 3, lines 91-96.**

**Comment 8:** Did you assess data distribution before representing it or selecting the best test?

**Response:** Thank you for this important statistical comment. We have revised the Statistical analysis section to clarify that data distribution was assessed before selecting statistical tests.

**Revision added:**
“The distribution of continuous variables was assessed using the Shapiro-Wilk test. Normally distributed variables were compared using the independent-samples t test, whereas non-normally distributed variables were compared using the Mann-Whitney U test. Categorical variables were compared using the chi-square test or Fisher’s exact test, as appropriate.”

**Location in revised manuscript: page 4, lines 137-141.**

**Comment 9:** Do the authors have the final histology of the included nodules? Otherwise, they should specify this among the limitations.

**Response:** Thank you for this important comment. All included nodules were confirmed by pathology. We have clarified this point in the Methods section.

**Revision added:**
“The final diagnosis of all pulmonary nodules was confirmed by pathological examination.”

**Location in revised manuscript: page 2, lines 74-75.**

**Comment 10:** “First, malignant nodules showed significantly higher early-phase MIBI uptake than benign nodules.” Ok, but please compare this considering the MIBI assessment for thyroid nodules that are considered suspicious/malignant when the early uptake is confirmed/increased in late acquisition.

**Response:** Thank you for this valuable suggestion. We have expanded the Discussion section to compare our findings with the established interpretation of 99mTc-MIBI imaging in thyroid nodules. We agree that in thyroid imaging, persistent or increased delayed uptake is often considered suspicious for malignancy. However, in pulmonary nodules, tracer kinetics may be influenced by different biological factors, including mitochondrial activity, perfusion, inflammatory background, and multidrug resistance-related efflux. Therefore, early uptake and delayed retention should be interpreted jointly rather than directly applying thyroid nodule criteria to pulmonary nodules.

**Revision added:**
“In thyroid nodule assessment, persistent or increased delayed 99mTc-MIBI uptake is often considered suspicious for malignancy. However, the kinetic behavior of 99mTc-MIBI in pulmonary nodules may differ because pulmonary lesions are affected by heterogeneous tumor biology, inflammatory changes, vascularity, mitochondrial activity, and multidrug resistance-related efflux mechanisms. In our cohort, malignant pulmonary nodules tended to show higher early uptake but lower retention index values, suggesting that early tracer accumulation and delayed washout may provide complementary information. Therefore, the interpretation of dual-phase 99mTc-MIBI SPECT/CT in pulmonary nodules should not simply follow thyroid nodule criteria, but should consider lesion-specific tracer kinetics.”

**Location in revised manuscript: Discussion, page 7, lines 227-235.**

**Comment 11:** I would suggest that authors be more cautious with their conclusion statements, as this is a single-centre, preliminary study.

**Response:** Thank you for this important suggestion. We agree that the conclusion in the original manuscript may have been too strong. We have revised the conclusion to emphasize the preliminary and single-center nature of the study and the need for external validation.

**Revised conclusion:**
“Dual-phase ^99^mTc-MIBI SPECT/CT semiquantitative parameters may provide useful auxiliary information for differentiating benign and malignant pulmonary nodules. A machine learning strategy based on simple and interpretable parameters showed preliminary diagnostic potential in this single-center retrospective cohort. However, these findings require further validation in larger, multicenter studies before clinical application.”

**Location in revised manuscript: Conclusion, page 9, lines 293–295.**

**Comment 12:** Please double-check all figure legends and titles, i.e., Figure 4 “Example text for the main manuscript”.

**Response:** Thank you for identifying this error. We apologize for the oversight. We have carefully checked and corrected all figure legends and titles. The template text in Figure 4 has been removed and replaced with a formal figure legend.

**Location in revised manuscript: figure legends, page 9, line 305. The Fig 2 and Fig 4 legends still require final correction.**

**Responses to Reviewer #2**

**Comment 1:** Overall, the manuscript is well written, and the methodology is clearly described and scientifically interesting. Nevertheless, the clinical utility of MIBI SPECT/CT in this context appears limited, and its potential role in clinical decision-making should be more clearly defined and discussed. I suggest to try to overcome some of the explicited limitations to ameliorate the manuscript.

**Response:** Thank you very much for your positive evaluation and constructive suggestion. We agree that the clinical role of ^99^mTc-MIBI SPECT/CT should be defined more clearly and cautiously. We have revised the Discussion section to clarify that ^99^mTc-MIBI SPECT/CT is not intended to replace histopathology or FDG PET/CT, but may serve as an auxiliary imaging tool for pulmonary nodule assessment, especially in settings where FDG PET/CT or advanced image analysis is not readily available.

**Revision added:**
In selected patients with indeterminate pulmonary nodules, especially in institutions where ^18^F-FDG PET/CT is unavailable, less accessible, or not routinely performed, semiquantitative^99^mTc-MIBI SPECT/CT parameters may provide additional functional information for risk stratification. The proposed machine learning model may help integrate simple imaging-derived kinetic parameters and clinical markers into a more interpretable assessment framework.

**Location in revised manuscript: Discussion, page 8, line 267-271.**

**Comment 2:** Line 91 and 93: the statement is repeated.

**Response:** Thank you for pointing this out. We have removed the repeated statement regarding the Declaration of Helsinki and revised this paragraph to make the description clearer and more concise.

**Location in revised manuscript: Study population, page 2.**

**Comment 3:** Line 91 and 95: prospective or retrospective study?

**Response:** Thank you for this important comment. We apologize for the inconsistency in the original manuscript. We have revised the manuscript to consistently describe the study as a retrospective study throughout the Abstract, Methods, and Ethics statement.

**Revision added:**
“This retrospective study was conducted in accordance with the Declaration of Helsinki and was approved by the Medical Ethics Committee of the Second Affiliated Hospital of Harbin Medical University (Approval No. KY 2020-213). Written informed consent was obtained from all participants.”

**Location in revised manuscript: abstract, page 1 line 18.**

**Comment 4:** Line 104-105: this is a result.

**Response:** Thank you for this suggestion. We agree that this sentence belongs to the Results section rather than the Methods section. We have moved the sentence describing the final number of included patients and the number of benign and malignant lesions from the Methods section to the Results section.

**Location in revised manuscript: Results, page 4, line 154.**

We again sincerely thank the Academic Editor and both reviewers for their careful assessment and constructive comments. These suggestions have helped us improve the clarity, methodological transparency, and clinical relevance of our manuscript. We hope that the revised manuscript is now suitable for publication in PLOS ONE.

Sincerely,

Yuzhuo Xing, M.D.
On behalf of all authors
