# Supplementary material for: Diagnostic performance of machine learning models based on dual-phase 99mTc-MIBI SPECT/CT semiquantitative parameters for differentiating benign and malignant pulmonary nodules
Source: PLoS One. 2026 Jul 6;21(7):e0353271. doi: 10.1371/journal.pone.0353271 (PMC13336150; doi:10.1371/journal.pone.0353271)
Supplement: Peer review history

# Peer review: Decision Letter 0

**DOI:** 10.1371/journal.pone.0353271.r001

**Authors:** Riccardo Laudicella

4 Jun 2026

PONE-D-26-17187

Diagnostic Performance of Machine Learning Models Based on Dual-Phase 99mTc-MIBI SPECT/CT Semiquantitative Parameters for Differentiating Benign and Malignant Pulmonary Nodules

PLOS One

Dear Dr. Xing,

Thank you for submitting your manuscript to PLOS ONE. After careful consideration, we feel that it has merit but does not fully meet PLOS ONE’s publication criteria as it currently stands. Therefore, we invite you to submit a revised version of the manuscript that addresses the points raised during the review process.

Please submit your revised manuscript by Jul 18 2026 11:59PM. If you will need more time than this to complete your revisions, please reply to this message or contact the journal office at plosone@plos.org. When you’re ready to submit your revision, log on to https://www.editorialmanager.com/pone/ and select the 'Submissions Needing Revision' folder to locate your manuscript file.

Please include the following items when submitting your revised manuscript:

- A letter that responds to each point raised by the academic editor and reviewer(s). You should upload this letter as a separate file labeled 'Response to Reviewers'.
- A marked-up copy of your manuscript that highlights changes made to the original version. You should upload this as a separate file labeled 'Revised Manuscript with Track Changes'.
- An unmarked version of your revised paper without tracked changes. You should upload this as a separate file labeled 'Manuscript'.

If you would like to make changes to your financial disclosure, please include your updated statement in your cover letter. Guidelines for resubmitting your figure files are available below the reviewer comments at the end of this letter.

If applicable, we recommend that you deposit your laboratory protocols in protocols.io to enhance the reproducibility of your results. Protocols.io assigns your protocol its own identifier (DOI) so that it can be cited independently in the future. For instructions see: https://journals.plos.org/plosone/s/submission-guidelines#loc-laboratory-protocols. Additionally, PLOS ONE offers an option for publishing peer-reviewed Lab Protocol articles, which describe protocols hosted on protocols.io. Read more information on sharing protocols at https://plos.org/protocols?utm_medium=editorial-email&utm_source=authorletters&utm_campaign=protocols.

As the corresponding author, your ORCID iD is verified in the submission system and will appear in the published article. PLOS supports the use of ORCID, and we encourage all coauthors to register for an ORCID iD and use it as well. Please encourage your coauthors to verify their ORCID iD within the submission system before final acceptance, as unverified ORCID iDs will not appear in the published article. *Only*  the individual author can complete the verification step; PLOS staff *cannot*  verify ORCID iDs on behalf of authors.

We look forward to receiving your revised manuscript.

Kind regards,

Riccardo Laudicella

Academic Editor

PLOS One

Journal Requirements:

When submitting your revision, we need you to address these additional requirements.

1. Please ensure that your manuscript meets PLOS ONE's style requirements, including those for file naming. The PLOS ONE style templates can be found at https://journals.plos.org/plosone/s/file?id=wjVg/PLOSOne_formatting_sample_main_body.pdf and https://journals.plos.org/plosone/s/file?id=ba62/PLOSOne_formatting_sample_title_authors_affiliations.pdf

2. Please provide a complete Data Availability Statement in the submission form, ensuring you include all necessary access information or a reason for why you are unable to make your data freely accessible. If your research concerns only data provided within your submission, please write "All data are in the manuscript and/or supporting information files" as your Data Availability Statement.

3. PLOS requires an ORCID iD for the corresponding author in Editorial Manager on papers submitted after December 6th, 2016. Please ensure that you have an ORCID iD and that it is validated in Editorial Manager. To do this, go to ‘Update my Information’ (in the upper left-hand corner of the main menu), and click on the Fetch/Validate link next to the ORCID field. This will take you to the ORCID site and allow you to create a new iD or authenticate a pre-existing iD in Editorial Manager.

4. Please amend your authorship list in your manuscript file to include author Gang Jin.

5. We note you have included a table to which you do not refer in the text of your manuscript. Please ensure that you refer to Table 2 in your text; if accepted, production will need this reference to link the reader to the Table.

6. Please include your tables and its captions as part of your main manuscript and remove the individual files.

If the reviewer comments include a recommendation to cite specific previously published works, please review and evaluate these publications to determine whether they are relevant and should be cited. There is no requirement to cite these works unless the editor has indicated otherwise.

[Note: HTML markup is below. Please do not edit.]

Reviewers' comments:

Reviewer's Responses to Questions

**Comments to the Author**

1. Is the manuscript technically sound, and do the data support the conclusions?

The manuscript must describe a technically sound piece of scientific research with data that supports the conclusions. Experiments must have been conducted rigorously, with appropriate controls, replication, and sample sizes. The conclusions must be drawn appropriately based on the data presented.

Reviewer #1: Partly

Reviewer #2: Yes

**********

2. Has the statistical analysis been performed appropriately and rigorously?

Reviewer #1: I Don't Know

Reviewer #2: Yes

**********

3. Have the authors made all data underlying the findings in their manuscript fully available?

The PLOS Data policy requires authors to make all data underlying the findings described in their manuscript fully available without restriction, with rare exception (please refer to the Data Availability Statement in the manuscript PDF file). The data should be provided as part of the manuscript or its supporting information, or deposited to a public repository. For example, in addition to summary statistics, the data points behind means, medians and variance measures should be available. If there are restrictions on publicly sharing data—e.g. participant privacy or use of data from a third party—those must be specified.

Reviewer #1: Yes

Reviewer #2: Yes

**********

4. Is the manuscript presented in an intelligible fashion and written in standard English?

PLOS ONE does not copyedit accepted manuscripts, so the language in submitted articles must be clear, correct, and unambiguous. Any typographical or grammatical errors should be corrected at revision, so please note any specific errors here.

Reviewer #1: Yes

Reviewer #2: Yes

**********

5. Review Comments to the Author

Please use the space provided to explain your answers to the questions above. You may also include additional comments for the author, including concerns about dual publication, research ethics, or publication ethics. (Please upload your review as an attachment if it exceeds 20,000 characters)

Reviewer #1: This paper assesses the diagnostic performance of different ML models using Dual-Phase 99mTc-MIBI SPECT/CT semiquantitative Parameters for the differential diagnosis of benign and malignant pulmonary nodules. Here are my suggestions:

In the whole paper, the authors should use precise EANM radiopharmaceutical nomenclature https://eanm.org/publications/guidelines/overview/nomenclature/

The authors should specify in the methods section which SPECT/CT scanner was used (i.e., vendor, model, and so on).

Did you use MIBI in a clinical trial, or is it normal practice in your country?

From which company did you purchase MIBI?

Please clarify whether the study was retrospective or prospective, detailing the EC ID number.

Please clarify which was the acquisition field of view for each patient (i.e., head-to-toe, only thorax,…).

Why did you acquire each step using a 20s interval per projection? How did you select this duration?

Did you assess data distribution before representing it or selecting the best test?

Do the authors have the final histology of the included nodules? Otherwise, they should specify this among the limitations.

“First, malignant nodules showed significantly higher early-phase MIBI uptake than benign nodules” Ok, but please compare this considering the MIBI assessment for thyroid nodules that are considered suspicious/malignant when the early uptake is confirmed/increased in late acquisition (see PMID 28733644).

I would suggest that authors be more cautious with their conclusion statements, as this is a single-centre, preliminary study.

Please double-check all figure legends and titles (i.e. figure 4 “Example text for the main manuscript”).

Reviewer #2: Dear Authors,

Overall, the manuscript is well written, and the methodology is clearly described and scientifically interesting. I particularly appreciated your transparent acknowledgment of the study’s multiple limitations. Nevertheless, the clinical utility of MIBI SPECT/CT in this context appears limited, and its potential role in clinical decision-making should be more clearly defined and discussed. I suggest to try to overcome some of the explicited limitations to ameliorate the manuscript.

Minor comments:

- line 91 and 93 the statement is repeated

- line 91 and 95: prospective or retrospective study?

- line 104-105 this is a result

Best regards

**********

6. PLOS authors have the option to publish the peer review history of their article (what does this mean?). If published, this will include your full peer review and any attached files.

If you choose “no”, your identity will remain anonymous but your review may still be made public.

**Do you want your identity to be public for this peer review?**  For information about this choice, including consent withdrawal, please see our Privacy Policy.

Reviewer #1: **Yes:**  Giorgia Ricciardello

Reviewer #2: No

**********

[NOTE: If reviewer comments were submitted as an attachment file, they will be attached to this email and accessible via the submission site. Please log into your account, locate the manuscript record, and check for the action link "View Attachments". If this link does not appear, there are no attachment files.]

To ensure your figures meet our technical requirements, please review our figure guidelines: https://journals.plos.org/plosone/s/figures

You may also use PLOS’s free figure tool, NAAS, to help you prepare publication quality figures: https://journals.plos.org/plosone/s/figures#loc-tools-for-figure-preparation.

NAAS will assess whether your figures meet our technical requirements by comparing each figure against our figure specifications.

# Author comment: Author response to Decision Letter 1

**DOI:** 10.1371/journal.pone.0353271.r002

9 Jun 2026

Response to Reviewers

Manuscript ID: PONE-D-26-17187

Title: Diagnostic Performance of Machine Learning Models Based on Dual-Phase 99mTc-MIBI SPECT/CT Semiquantitative Parameters for Differentiating Benign and Malignant Pulmonary Nodules

Dear Editor and Reviewers,

We sincerely thank the Academic Editor and the reviewers for their careful evaluation of our manuscript and for the constructive and helpful comments. We have revised the manuscript thoroughly according to the comments. The major revisions include clarification of the study design and ethics approval, supplementation of detailed SPECT/CT acquisition information, standardization of the radiopharmaceutical nomenclature, clarification of the clinical research use and source of 99mTc-MIBI, improvement of the statistical methods, revision of the discussion and conclusion to avoid overstatement, correction of figure legends, and formatting changes according to PLOS ONE requirements.

Comment 1: Please ensure that your manuscript meets PLOS ONE's style requirements, including those for file naming.

Response: Thank you for this reminder. We have carefully revised the manuscript according to the PLOS ONE style requirements, including the title page, author information, main text, tables, figure legends, and file naming.

Comment 2: Please provide a complete Data Availability Statement in the submission form, ensuring you include all necessary access information or a reason for why you are unable to make your data freely accessible.

Response: Thank you for this comment. We have added a complete Data Availability Statement in the submission system and in the revised manuscript. Because the data contain potentially identifying patient information, the raw clinical and imaging data cannot be made publicly available due to patient privacy and ethical restrictions. Data are available from the corresponding author upon reasonable request and with approval from the Medical Ethics Committee of the Second Affiliated Hospital of Harbin Medical University.

Revised Data Availability Statement:

“The datasets generated and/or analyzed during the current study are not publicly available because they contain potentially identifying patient information. Data are available from the corresponding author upon reasonable request and with approval from the Medical Ethics Committee of the Second Affiliated Hospital of Harbin Medical University.”

Comment 3: PLOS requires an ORCID iD for the corresponding author in Editorial Manager.

Response: Thank you for this reminder. The corresponding author has verified the ORCID iD in the Editorial Manager system.

Comment 4: Please amend your authorship list in your manuscript file to include author Gang Jin.

Response: Thank you for pointing this out. We have amended the authorship list to include Gang Jin in the revised manuscript. We have also updated the author contribution statement accordingly.

Revision made:

“Administrative support: Xin Zhou and Gang Jin.”

Comment 5: We note you have included a table to which you do not refer in the text of your manuscript. Please ensure that you refer to Table 2 in your text; if accepted, production will need this reference to link the reader to the Table.

Response: Thank you for this helpful comment. We have added an explicit reference to Table 2 in the Results section.

Revision added:

“The comparison of semiquantitative parameters between benign and malignant pulmonary nodules is shown in Table 2.”

Comment 6: Please include your tables and its captions as part of your main manuscript and remove the individual files.

Response: Thank you for this reminder. We have included all tables and their captions in the main manuscript and removed the individual table files during resubmission.

Responses to Reviewer #1

Comment 1: In the whole paper, the authors should use precise EANM radiopharmaceutical nomenclature.

Response: Thank you for this important suggestion. We have carefully checked the entire manuscript and standardized the radiopharmaceutical nomenclature according to the EANM recommendations. The term 99mTc-MIBI has been used consistently throughout the manuscript, including the title, abstract, main text, tables, and figure legends.

Comment 2: The authors should specify in the methods section which SPECT/CT scanner was used, i.e., vendor, model, and so on.

Response: Thank you for this comment. We agree that detailed scanner information is important for reproducibility. We have added the vendor and model of the SPECT/CT system in the Methods section.

Revision added:

“All SPECT/CT examinations were performed using a Philips Precedence 6-slice hybrid SPECT/CT system (Philips Nuclear Medicine, Inc., Milpitas, CA, USA) equipped with low-energy high-resolution collimators.”

Comment 3: Did you use MIBI in a clinical trial, or is it normal practice in your country?

Response: Thank you for this question. We have clarified this point in the revised Methods section. In this study, 99mTc-MIBI SPECT/CT was performed under an ethics-approved clinical research protocol for the evaluation of pulmonary nodules. It was not a therapeutic clinical trial.

Revision added:

“Dual-phase 99mTc-MIBI SPECT/CT was performed under an ethics-approved clinical research protocol for pulmonary nodule assessment and was not conducted as a therapeutic clinical trial.”

Comment 4: From which company did you purchase MIBI?

Response: Thank you for this comment. We have added the source of 99mTc-MIBI in the Methods section.

Revision added:

“99mTc-MIBI was purchased from Jiangyuan Pharmaceutical Factory of Wuxi Jiangyuan Industrial Technology and Trade Co., Ltd. (Wuxi, China).”

Comment 5: Please clarify whether the study was retrospective or prospective, detailing the EC ID number.

Response: Thank you for pointing out this important inconsistency. We apologize for the unclear description in the original manuscript. We have now revised the manuscript to consistently describe the study as a retrospective study throughout the Abstract, Methods, and Ethics statement. We have also provided the ethics approval number.

Revision added:

“This retrospective study was conducted in accordance with the Declaration of Helsinki and was approved by the Medical Ethics Committee of the Second Affiliated Hospital of Harbin Medical University (Approval No. KY 2020-213).

Comment 6: Please clarify which was the acquisition field of view for each patient, i.e., head-to-toe, only thorax, and so on.

Response: Thank you for this helpful suggestion. We have clarified the acquisition field of view in the Methods section.

Revision added:

“The acquisition field of view covered only the thorax/chest region, including the pulmonary lesion and adjacent thoracic structures.”

Comment 7: Why did you acquire each step using a 20 s interval per projection? How did you select this duration?

Response: Thank you for this comment. We have added an explanation in the Methods section. The acquisition time of 20 s per projection was selected according to the routine imaging protocol of our center. This duration was used to balance sufficient count statistics and image quality with an acceptable total acquisition time for patients.

Revision added:

“The acquisition time of 20 s per projection was selected according to the standard imaging protocol of our center, aiming to obtain sufficient count statistics and acceptable image quality while avoiding an unnecessarily long acquisition time for patients.”

Comment 8: Did you assess data distribution before representing it or selecting the best test?

Response: Thank you for this important statistical comment. We have revised the Statistical analysis section to clarify that data distribution was assessed before selecting statistical tests.

Revision added:

“The distribution of continuous variables was assessed using the Shapiro-Wilk test. Normally distributed variables were compared using the independent-samples t test, whereas non-normally distributed variables were compared using the Mann-Whitney U test. Categorical variables were compared using the chi-square test or Fisher’s exact test, as appropriate.”

Comment 9: Do the authors have the final histology of the included nodules? Otherwise, they should specify this among the limitations.

Response: Thank you for this important comment. All included nodules were confirmed by pathology. We have clarified this point in the Methods section.

Revision added:

“The final diagnosis of all pulmonary nodules was confirmed by pathological examination.”

Comment 10: “First, malignant nodules showed significantly higher early-phase MIBI uptake than benign nodules.” Ok, but please compare this considering the MIBI assessment for thyroid nodules that are considered suspicious/malignant when the early uptake is confirmed/increased in late acquisition.

Response: Thank you for this valuable suggestion. We have expanded the Discussion section to compare our findings with the established interpretation of 99mTc-MIBI imaging in thyroid nodules. We agree that in thyroid imaging, persistent or increased delayed uptake is often considered suspicious for malignancy. However, in pulmonary nodules, tracer kinetics may be influenced by different biological factors, including mitochondrial activity, perfusion, inflammatory background, and multidrug resistance-related efflux. Therefore, early uptake and delayed retention should be interpreted jointly rather than directly applying thyroid nodule criteria to pulmonary nodules.

Revision added:

“In thyroid nodule assessment, persistent or increased delayed 99mTc-MIBI uptake is often considered suspicious for malignancy. However, the kinetic behavior of 99mTc-MIBI in pulmonary nodules may differ because pulmonary lesions are affected by heterogeneous tumor biology, inflammatory changes, vascularity, mitochondrial activity, and multidrug resistance-related efflux mechanisms. In our cohort, malignant pulmonary nodules tended to show higher early uptake but lower retention index values, suggesting that early tracer accumulation and delayed washout may provide complementary information. Therefore, the interpretation of dual-phase 99mTc-MIBI SPECT/CT in pulmonary nodules should not simply follow thyroid nodule criteria, but should consider lesion-specific tracer kinetics.”

Comment 11: I would suggest that authors be more cautious with their conclusion statements, as this is a single-centre, preliminary study.

Response: Thank you for this important suggestion. We agree that the conclusion in the original manuscript may have been too strong. We have revised the conclusion to emphasize the preliminary and single-center nature of the study and the need for external validation.

Revised conclusion:

“Dual-phase 99mTc-MIBI SPECT/CT semiquantitative parameters may provide useful auxiliary information for differentiating benign and malignant pulmonary nodules. A machine learning strategy based on simple and interpretable parameters showed preliminary diagnostic potential in this single-center retrospective cohort. However, these findings require further validation in larger, multicenter studies before clinical application.”

Comment 12: Please double-check all figure legends and titles, i.e., Figure 4 “Example text for the main manuscript”.

Response: Thank you for identifying this error. We apologize for the oversight. We have carefully checked and corrected all figure legends and titles. The template text in Figure 4 has been removed and replaced with a formal figure legend.

Responses to Reviewer #2

Comment 1: Overall, the manuscript is well written, and the methodology is clearly described and scientifically interesting. Nevertheless, the clinical utility of MIBI SPECT/CT in this context appears limited, and its potential role in clinical decision-making should be more clearly defined and discussed. I suggest to try to overcome some of the explicited limitations to ameliorate the manuscript.

Response: Thank you very much for your positive evaluation and constructive suggestion. We agree that the clinical role of 99mTc-MIBI SPECT/CT should be defined more clearly and cautiously. We have revised the Discussion section to clarify that 99mTc-MIBI SPECT/CT is not intended to replace histopathology or FDG PET/CT, but may serve as an auxiliary imaging tool for pulmonary nodule assessment, especially in settings where FDG PET/CT or advanced image analysis is not readily available.

Revision added:

In selected patients with indeterminate pulmonary nodules, especially in institutions where 18F-FDG PET/CT is unavailable, less accessible, or not routinely performed, semiquantitative99mTc-MIBI SPECT/CT parameters may provide additional functional information for risk stratification. The proposed machine learning model may help integrate simple imaging-derived kinetic parameters and clinical markers into a more interpretable assessment framework.

Comment 2: Line 91 and 93: the statement is repeated.

Response: Thank you for pointing this out. We have removed the repeated statement regarding the Declaration of Helsinki and revised this paragraph to make the description clearer and more concise.

Comment 3: Line 91 and 95: prospective or retrospective study?

Response: Thank you for this important comment. We apologize for the inconsistency in the original manuscript. We have revised the manuscript to consistently describe the study as a retrospective study throughout the Abstract, Methods, and Ethics statement.

Revision added:

“This retrospective study was conducted in accordance with the Declaration of Helsinki and was approved by the Medical Ethics Committee of the Second Affiliated Hospital of Harbin Medical University (Approval No. KY 2020-213). Written informed consent was obtained from all participants.”

Comment 4: Line 104-105: this is a result.

Response: Thank you for this suggestion. We agree that this sentence belongs to the Results section rather than the Methods section. We have moved the sentence describing the final number of included patients and the number of benign and malignant lesions from the Methods section to the Results section.

We again sincerely thank the Academic Editor and both reviewers for their careful assessment and constructive comments. These suggestions have helped us improve the clarity, methodological transparency, and clinical relevance of our manuscript. We hope that the revised manuscript is now suitable for publication in PLOS ONE.

Sincerely,

Yuzhuo Xing, M.D.

On behalf of all authors

Attachment Submitted filename: Response to Reviewers_with locations.docx

# Peer review: Decision Letter 1

**DOI:** 10.1371/journal.pone.0353271.r003

**Authors:** Riccardo Laudicella, Riccardo Laudicella

22 Jun 2026

Diagnostic Performance of Machine Learning Models Based on Dual-Phase 99mTc-MIBI SPECT/CT Semiquantitative Parameters for Differentiating Benign and Malignant Pulmonary Nodules

PONE-D-26-17187R1

Dear Dr. Xing

We’re pleased to inform you that your manuscript has been judged scientifically suitable for publication and will be formally accepted for publication once it meets all outstanding technical requirements.

Within one week, you’ll receive an e-mail detailing the required amendments. When these have been addressed, you’ll receive a formal acceptance letter and your manuscript will be scheduled for publication.

An invoice will be generated when your article is formally accepted. Please note, if your institution has a publishing partnership with PLOS and your article meets the relevant criteria, all or part of your publication costs will be covered. Please make sure your user information is up-to-date by logging into Editorial Manager at Editorial Manager® and clicking the ‘Update My Information' link at the top of the page. For questions related to billing, please contact billing support.

If your institution or institutions have a press office, please notify them about your upcoming paper to help maximize its impact. If they’ll be preparing press materials, please inform our press team as soon as possible -- no later than 48 hours after receiving the formal acceptance. Your manuscript will remain under strict press embargo until 2 pm Eastern Time on the date of publication. For more information, please contact onepress@plos.org.

Kind regards,

Riccardo Laudicella

Academic Editor

PLOS One

Additional Editor Comments (optional):

Reviewers' comments:

Reviewer's Responses to Questions

**Comments to the Author**

1. If the authors have adequately addressed your comments raised in a previous round of review and you feel that this manuscript is now acceptable for publication, you may indicate that here to bypass the “Comments to the Author” section, enter your conflict of interest statement in the “Confidential to Editor” section, and submit your "Accept" recommendation.

Reviewer #1: All comments have been addressed

Reviewer #2: All comments have been addressed

**********

2. Is the manuscript technically sound, and do the data support the conclusions?

The manuscript must describe a technically sound piece of scientific research with data that supports the conclusions. Experiments must have been conducted rigorously, with appropriate controls, replication, and sample sizes. The conclusions must be drawn appropriately based on the data presented.

Reviewer #1: (No Response)

Reviewer #2: Yes

**********

3. Has the statistical analysis been performed appropriately and rigorously?

Reviewer #1: (No Response)

Reviewer #2: Yes

**********

4. Have the authors made all data underlying the findings in their manuscript fully available?

The PLOS Data policy requires authors to make all data underlying the findings described in their manuscript fully available without restriction, with rare exception (please refer to the Data Availability Statement in the manuscript PDF file). The data should be provided as part of the manuscript or its supporting information, or deposited to a public repository. For example, in addition to summary statistics, the data points behind means, medians and variance measures should be available. If there are restrictions on publicly sharing data—e.g. participant privacy or use of data from a third party—those must be specified.

Reviewer #1: (No Response)

Reviewer #2: Yes

**********

5. Is the manuscript presented in an intelligible fashion and written in standard English?

PLOS ONE does not copyedit accepted manuscripts, so the language in submitted articles must be clear, correct, and unambiguous. Any typographical or grammatical errors should be corrected at revision, so please note any specific errors here.

Reviewer #1: (No Response)

Reviewer #2: Yes

**********

6. Review Comments to the Author

Please use the space provided to explain your answers to the questions above. You may also include additional comments for the author, including concerns about dual publication, research ethics, or publication ethics. (Please upload your review as an attachment if it exceeds 20,000 characters)

Reviewer #1: (No Response)

Reviewer #2: Dear Authors,

Thank you for addressing the comments and revising the manuscript in accordance with the suggestions provided. The manuscript is now more comprehensive and precise.

Best regards

**********

7. PLOS authors have the option to publish the peer review history of their article (what does this mean?). If published, this will include your full peer review and any attached files.

If you choose “no”, your identity will remain anonymous but your review may still be made public.

**Do you want your identity to be public for this peer review?**  For information about this choice, including consent withdrawal, please see our Privacy Policy.

Reviewer #1: **Yes:**  Giorgia Ricciardello

Reviewer #2: No

**********

# Editor report: Acceptance letter

**DOI:** 10.1371/journal.pone.0353271.r004

**Authors:** Riccardo Laudicella, Riccardo Laudicella

PONE-D-26-17187R1

PLOS One

Dear Dr. Xing,

I'm pleased to inform you that your manuscript has been deemed suitable for publication in PLOS One. Congratulations! Your manuscript is now being handed over to our production team.

At this stage, our production department will prepare your paper for publication. This includes ensuring the following:

* All references, tables, and figures are properly cited

* All relevant supporting information is included in the manuscript submission,

* There are no issues that prevent the paper from being properly typeset

You will receive further instructions from the production team, including instructions on how to review your proof when it is ready. Please keep in mind that we are working through a large volume of accepted articles, so please give us a few days to review your paper and let you know the next and final steps.

Lastly, if your institution or institutions have a press office, please let them know about your upcoming paper now to help maximize its impact. If they'll be preparing press materials, please inform our press team within the next 48 hours. Your manuscript will remain under strict press embargo until 2 pm Eastern Time on the date of publication. For more information, please contact onepress@plos.org.

You will receive an invoice from PLOS for your publication fee after your manuscript has reached the completed accept phase. If you receive an email requesting payment before acceptance or for any other service, this may be a phishing scheme. Learn how to identify phishing emails and protect your accounts at https://explore.plos.org/phishing.

If we can help with anything else, please email us at customercare@plos.org.

Thank you for submitting your work to PLOS ONE and supporting open access.

Kind regards,

PLOS ONE Editorial Office Staff

on behalf of

Dr. Riccardo Laudicella

Academic Editor

PLOS One
